# Contributions of *Streptococcus mutans* Cnm and PA antigens to aggravation of non-alcoholic steatohepatitis in mice

**DOI:** 10.1038/srep36886

**Published:** 2016-11-11

**Authors:** Shuhei Naka, Rina Hatakeyama, Yukiko Takashima, Michiyo Matsumoto-Nakano, Ryota Nomura, Kazuhiko Nakano

**Affiliations:** 1 Department of Pediatric Dentistry, Division of Oral Infection and Disease Control, Osaka University Graduate School of Dentistry, Suita, Osaka, Japan; 2 Department of Pediatric Dentistry, Okayama University Graduate School of Medicine, Dentistry and Pharmaceutical Sciences, Okayama, Japan

## Abstract

*Streptococcus mutans*, a major pathogen of dental caries, can cause infective endocarditis after invading the bloodstream. Recently, intravenous administration of specific *S. mutans* strains was shown to aggravate non-alcoholic steatohepatitis (NASH) in a mouse model fed a high-fat diet. Here, we investigated the mechanism of this aggravation in a NASH mouse model by focusing on the *S. mutans* cell surface collagen-binding protein (Cnm) and the 190-kDa protein antigen (PA). Mice that were intravenously administered a *S. mutans* strain with a defect in Cnm (TW871CND) or PA (TW871PD) did not show clinical or histopathological signs of NASH aggravation, in contrast to those administered the parent strain TW871. The immunochemical analyses demonstrated higher levels of interferon-γ and metallothionein expression in the TW871 group than in the TW871CND and TW871PD groups. Analysis of bacterial affinity to cultured hepatic cells in the presence of unsaturated fatty acids revealed that the incorporation rate of TW871 was significantly higher than those of TW871CND and TW871PD. Together, our results suggest that Cnm and PA are important cell surface proteins for the NASH aggravation caused by *S. mutans* adhesion and affinity for hepatic cells.

*Streptococcus mutans*, a major pathogen of dental caries, is occasionally isolated from the blood of patients with infective endocarditis^1^. Notably, the cell surface collagen-binding protein known as Cnm, which contributes to the adhesion to and invasion of arterial and venous endothelial cells, is an important factor for the development of infective endocarditis^2^,^3^. Furthermore, our recent studies indicate that intravenous administration of *S. mutans* strains with Cnm expression may be associated with an aggravation of cerebral haemorrhage and inflammatory bowel disease^4^,^5^. Some Cnm-positive strains of *S. mutans* lack expression of the 190-kDa cell surface protein antigen (PA), also termed SpaP, P1, or antigen I/II, which is associated with sucrose-independent initial adhesion to tooth surfaces^6^. One study found that a Cnm^+^/PA^+^ strain of *S. mutans* contributed to the aggravation of non-alcoholic steatohepatitis (NASH) in mice but that neither the tested Cnm^+^/PA^−^ strain nor the tested Cnm^−^/PA^−^ strain did so^7^, suggesting that Cnm and PA could be important cell surface proteins for *S. mutans*-associated aggravation of NASH. In that study, microarray analysis findings additionally observed that the levels of interferon (IFN)γ and metallothionein mRNA expression in NASH liver tissues are elevated compared with those in healthy controls^7^. However, the precise mechanism for the contribution of Cnm and PA to *S. mutans*-associated aggravation of NASH remains to be elucidated.

The “two-hit theory of NASH development” is widely accepted, which claims that fatty liver is the first stage leading to NASH, followed by inflammation as a second hit^8^. Fatty liver is defined as a condition in which the accumulation of triacylglycerol, a major component of neutral fat, is greater than 5% in liver tissues^9^. Triacylglycerol is composed of a glycerol bound to three fatty acids, which are mainly classified as saturated or unsaturated fatty acids^10^. Palmitic acid, a saturated fatty acid that is important for lipid formation in humans, is solid at room temperature and considered to be a possible factor in atherosclerosis, cerebral infarction, and fatty liver diseases^11^,^12^,^13^. Additionally, palmitic acid has a chemical structure similar to that of hexadecane^14^, which is mainly used in the cellular hydrophobicity assays that are commonly performed for studying the virulence of dental caries caused by *S. mutans*^15^. Oleic acid, an unsaturated fatty acid that is an important energy source for humans, is liquid at room temperature and thus considered to be less harmful to humans than palmitic acid^16^,^17^. However, the association between saturated and/or unsaturated fatty acids and NASH aggravation remains to be elucidated.

Based on these factors, we hypothesised that Cnm^+^/PA^+^ *S. mutans* strains could incorporate into liver tissues that had accumulated fatty acids, leading to the aggravation of NASH. To examine this possible mechanism of *S. mutans*-associated aggravation of NASH, we assessed the NASH aggravation in a NASH mouse model following the administration of Cnm- or PA-defective isogenic mutant strains or their complemented mutant strains. The affinity of bacterial cells for various fatty acids was then evaluated using protocols based on cellular hydrophobicity assays and focusing on the *S. mutans* bacterial cell surface antigens Cnm and PA. Additionally, we investigated the interaction of bacterial cells with various types of fatty acids using cultured hepatic cells to evaluate differences in their fat accumulation.

## Results

### *S. mutans* strains induced aggravation of NASH in model mice

**Figure 1:**
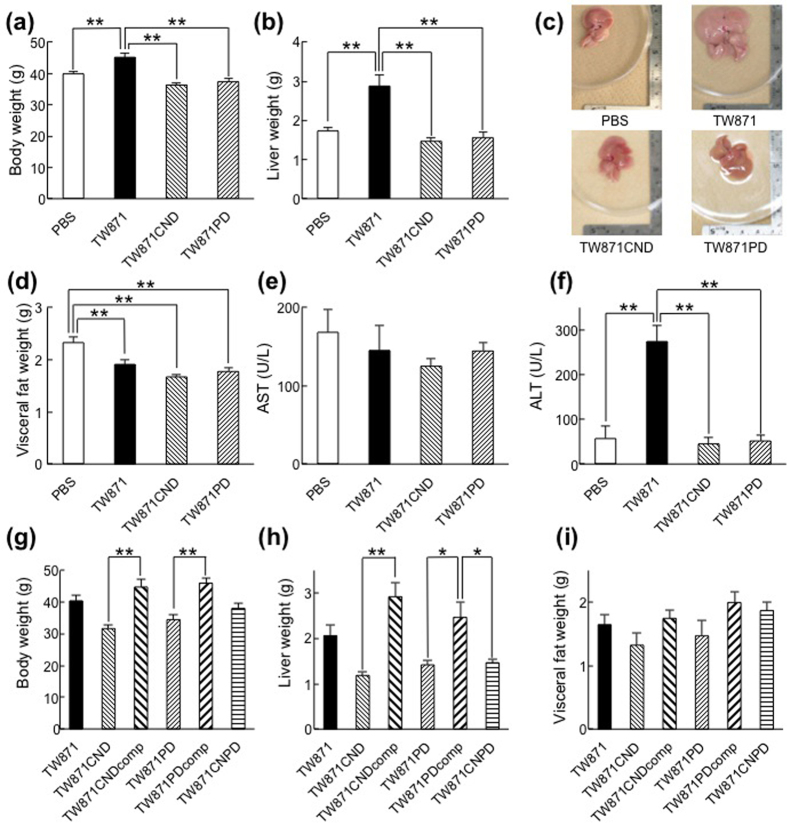
Evaluation of TW871 Cnm and PA in regard to the aggravation of NASH. (**a**–**f**) After male 6-week-old C57BL/6 J male mice were fed a high-fat diet (HFD) for 4 weeks, they were infected with 1 × 10^7^ CFU of *S. mutans* TW871, TW871CND, or TW871PD or were mock-infected with PBS. Twelve weeks after infection, the mice were euthanised (16 weeks after initiating feeding with a HFD), and their NASH characteristics were evaluated. Body (**a**) and liver (**b**) weights in each group. Representative liver specimens from each group (**c**). Visceral fat weight (**d**) and serum levels of AST (**e**) and ALT (**f)** in each group. (**g**–**i**) The same experiments were then performed using groups of mice infected with TW871, TW871CND, TW871CNDcomp, TW871PD, TW871PDcomp, or TW871CNPD. Body (**g**) liver (**h**) and visceral fat (**i**) weights in each group. Each column represents the mean ± SEM from 11–17 different animals. Statistical significance was determined using Bonferroni’s method after analysis of variance (ANOVA). *p < 0.05, **p < 0.01.

The results of our NASH model mouse experiments in Experiment 1 demonstrate that infection with a strain of *S. mutans* that is defective in Cnm (TW871CND) or in PA (TW871PD) resulted in reduced body and liver weights as compared with infection with the parent strain TW871 (Fig. 1a–c). There were no significant differences in the weights of visceral fat among the TW871-, TW871CND-, and TW871PD-infected mice, all of which were significantly lower than the weight of visceral fat in phosphate-buffered saline (PBS)-treated mice (Fig. 1d). Furthermore, serum aspartate aminotransferase (AST) values for these groups were similar, but serum alanine aminotransaminase (ALT) values in the TW871-infected mice were significantly higher than those in the TW871CND- and TW871PD-infected mice (Fig. 1e,f), demonstrating that only the group infected with TW871 developed typical NASH symptoms.

In Experiment 2, analyses of the complemented mutant strains of Cnm (TW871CNDcomp) and PA (TW871PDcomp) demonstrate that body and liver weights in the groups of mice infected with either of these strains were recovered to levels similar to those of mice infected with TW871 (Fig. 1g,h). In contrast, the mice infected with the Cnm and PA double knockout strain (TW871CNPD) showed similar characteristics (body and liver weight) as those infected with TW871CND or TW871PD. There were no significant differences in the weight of visceral fat among any of the groups (Fig. 1i).

### Bacterial clearance in the NASH mouse model

**Figure 2:**
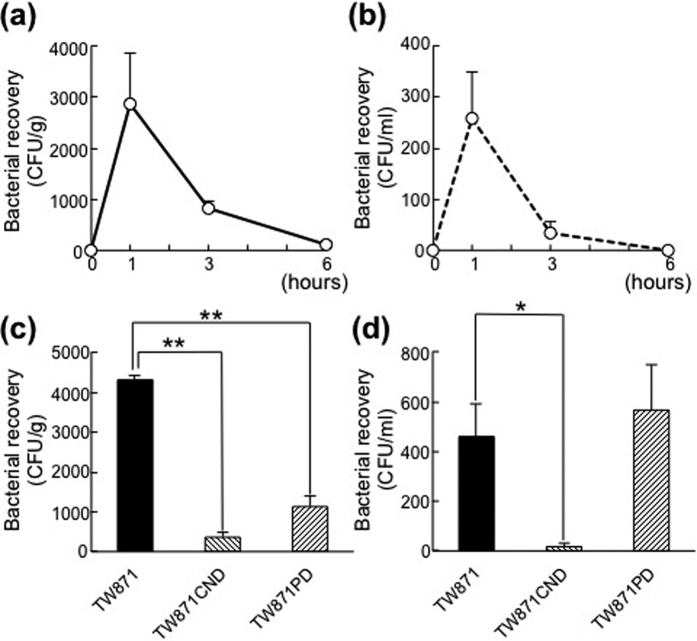
Transitional changes in bacterial recovery from blood and liver. (**a**,**b**) C57BL/6 J male mice were infected with 1 × 10^7^ CFU of *S. mutans* TW871, and liver and blood specimens were obtained from the mice just before infection and 1, 3, and 6 h after infection (n = 5–6 each). These specimens were diluted 10-fold with sterile PBS and cultured on Mitis-Salivarius agar plates containing bacitracin. The numbers of visible colonies were counted to determine the bacterial numbers from each specimen, which were standardised by the liver weight (g) or blood amount (ml). The recovered strains were confirmed to be *S. mutans* using a PCR-based method with a *S. mutans*–specific set of primers. The amounts of bacterial recovery in liver (**a**) and blood (**b**) are expressed as mean values ± SEM. (**c–d**) The same experiments were then performed using groups of mice infected with TW871, TW871CND, or TW871PD, and liver and blood specimens were obtained from the mice 1 h after infection (n = 5–6 each). The amounts of bacterial recovery in the liver (**c**) and blood (**d**) are expressed as mean values ± SEM. Statistical significance was determined using Bonferroni’s method after analysis of variance (ANOVA). *p < 0.05, **p < 0.01.

Bacterial recovery analyses of mice infected with TW871 demonstrate that approximately 3,000 and 800 colony-forming units (CFU) per gram weight were present in the liver tissue at 1 h and 3 h after infection, respectively, whereas few bacterial strains (approximately 100 CFU per gram) were recovered at 6 h after infection (Fig. 2a). For the blood specimens, approximately 250 and 30 CFU/ml blood were isolated from the specimens taken at 1 h and 3 h after infection, respectively. No bacterial strains were recovered at 6 h after infection (Fig. 2b). The arbitrarily primed (AP)-PCR analyses demonstrate that the fingerprinting patterns of the bacteria recovered from the liver and blood specimens were completely consistent with that of the strain used to infect the mice, TW871 (Fig. S1). When analysing the bacterial localisation in mice 1 h after infection with TW871, the administered bacteria could be recovered from the liver, blood, and spleen of all five mice, whereas the tested bacteria could be recovered from the heart and kidney in only two out of five mice (Table S1). Additionally, no tested bacteria could be recovered from the lung, pancreas, or colon of any of the infected mice. We then evaluated the bacterial clearance 1 h after infection with TW871CND and TW871PD as compared with that following infection with TW871. We found that the amount of bacterial recovery in the liver was significantly lower in the groups of mice infected with TW871CND or TW871PD than that in the group of mice infected with TW871 (Fig. 2c). However, compared with the bacterial clearance of TW871 in the blood, that of TW871PD was similar and that of TW871CND was higher (Fig. 2d).

### Histopathological appearance in the NASH mouse model

**Figure 3:**
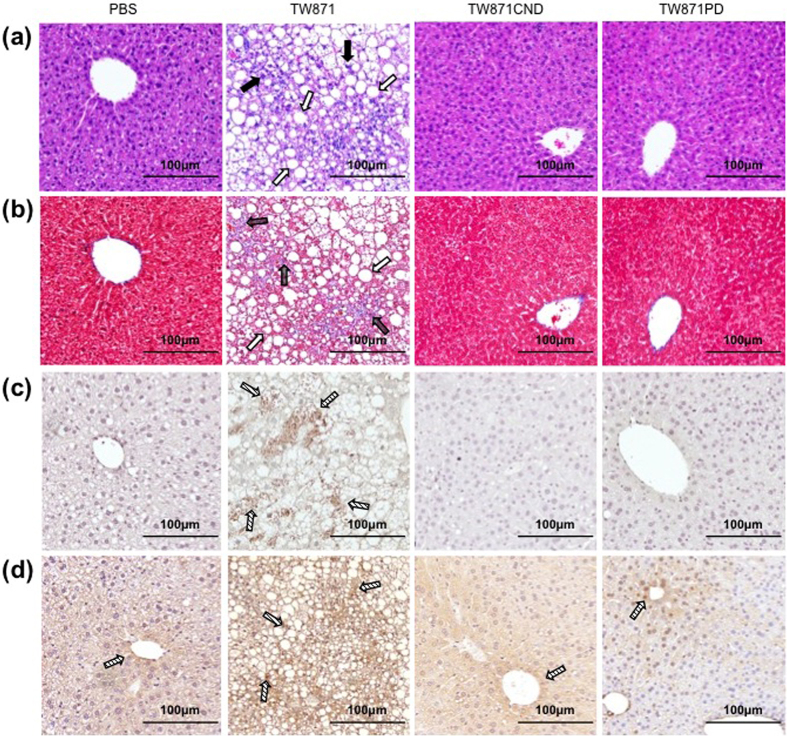
Representative histopathological appearance of liver tissues. After being fed with a HFD for 4 weeks, male, 6-week-old C57BL/6 J mice were infected with 1 × 10^7^ CFU of *S. mutans* TW871, TW871CND, or TW871PD or were mock-infected with PBS after feeding 6-week-old mice with a HFD for four weeks. Twelve weeks after infection, the mice (n = 11–17) were euthanised (16 weeks after initiating feeding with a HFD), and the NASH characteristics were evaluated. Representative images from each group of sections stained with haematoxylin-eosin (**a**) or Masson’s trichrome (**b**) staining or immunostained for IFNγ (**c**) or metallothionein (**d**). The closed and open arrows in panel (**a**) indicate inflammatory cells and adipose deposition, respectively. The dark and open arrows in panel (**b**) indicate fibrosis and adipose deposition, respectively. The arrows in panels (**c**,**d**) indicate a positive immunostaining reaction.

Histopathological and immunochemical analyses were conducted on liver tissues extirpated from NASH mice infected with TW871, TW871CND, or TW871PD or mock-infected with PBS (Fig. 3). Liver specimens from the TW871-infected group displayed typical NASH characteristics, such as prominent infiltration of inflammatory cells and adipocellular deposition (Fig. 3a,b). In contrast, no NASH characteristics were observed in the TW871CND- or TW871PD-infected mice. Additionally, immunostaining analyses showed that the levels of IFNγ and metallothionein expression were elevated in liver specimens from the TW871-infected mice, while the expression levels of these cytokines were very low in the samples from mice infected with TW871CND- or TW871PD (Fig. 3c,d).

### Affinity of cultured hepatic cells for fatty acids

**Figure 4:**
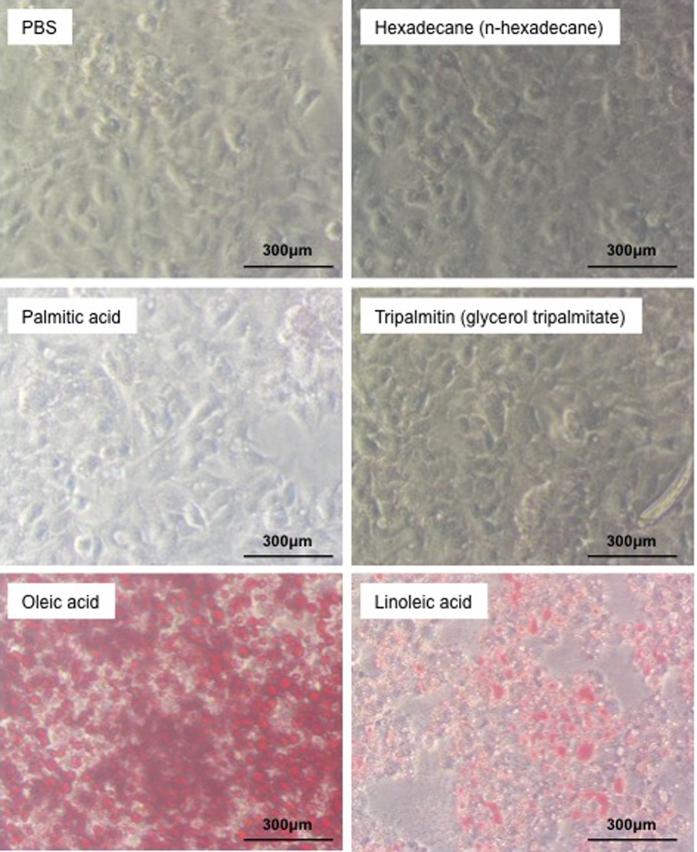
Affinity of cultured hepatic cells for saturated and unsaturated fatty acids. HepG2 cells were cultured with PBS, hexadecane, saturated fatty acids (palmitic acid or tripalmitin), or unsaturated fatty acids (oleic acid or linoleic acid) and stained with Oil Red O. The red staining indicates the accumulation of neutral fat in the HepG2 cells. The experiments were performed three times. Representative images are shown.

The results from Oil Red O staining, in which red staining indicates neutral fat accumulation of HepG2 cells cultured with fatty acids demonstrate that these cells react with unsaturated fatty acids, such as oleic acid and linoleic acid, but not with saturated fatty acids, such as palmitic acid and tripalmitin. The levels of Oil Red O staining in cells cultured with saturated fatty acids are similar to those in the cells cultured with PBS or hexadecane (Fig. 4). The accumulation of neutral fat in HepG2 cells was more prominent in cells treated with oleic acid than that in cells treated with as linoleic acid.

### Incorporation of fatty acids by *S. mutans* strains

**Figure 5:**
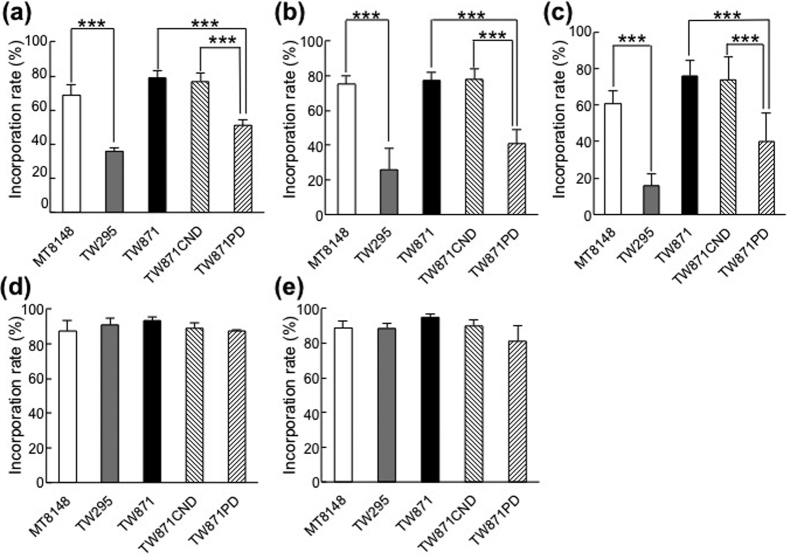
Incorporation by *S. mutans* strains of saturated or unsaturated fatty acids. The incorporation by MT8148, TW295, TW871, TW871CND, and TW871PD of (**a**) hexadecane, (**b**) oleic acid, (**c**) linoleic acid, (**d**) palmitic acid, and (**e**) tripalmitin were evaluated. The incorporation rate was calculated as follows: [1 − OD_550_ (aqueous phase of the tube containing cell suspensions with added n-hexadecane or one of the tested fatty acids)/OD_550_ (aqueous phase of the tube containing only cell suspensions) × 100 (%)]. The results are shown as the mean ± SD from five independent experiments. Statistical significance was determined using Bonferroni’s method after ANOVA. ***p < 0.001.

The bacterial incorporation of each fatty acid was evaluated using various *S. mutans* strains. The rates of hexadecane incorporation by PA^+^ strains, TW871, TW871CND, and a standard oral strain used in many studies, MT8148, were similar to one another, but these incorporation rates were all significantly higher than those of PA^−^ strains, TW295 and TW871PD (*p* < 0.001) (Fig. 5a). The incorporation rates of the tested unsaturated fatty acids, oleic acid and linoleic acid, by TW871, TW871CND, and MT8148 cells were similar to one another, though these were all significantly higher than incorporation rates of unsaturated fatty acids by TW295 and TW871PD (*p* < 0.001) (Fig. 5b,c). In contrast, none of the examined strains showed significant differences in their incorporation levels of saturated fatty acids, such as palmitic acid and tripalmitin (Fig. 5d,e).

### Bacterial adhesion to hepatic cells in the presence or absence of unsaturated fatty acids

**Figure 6:**
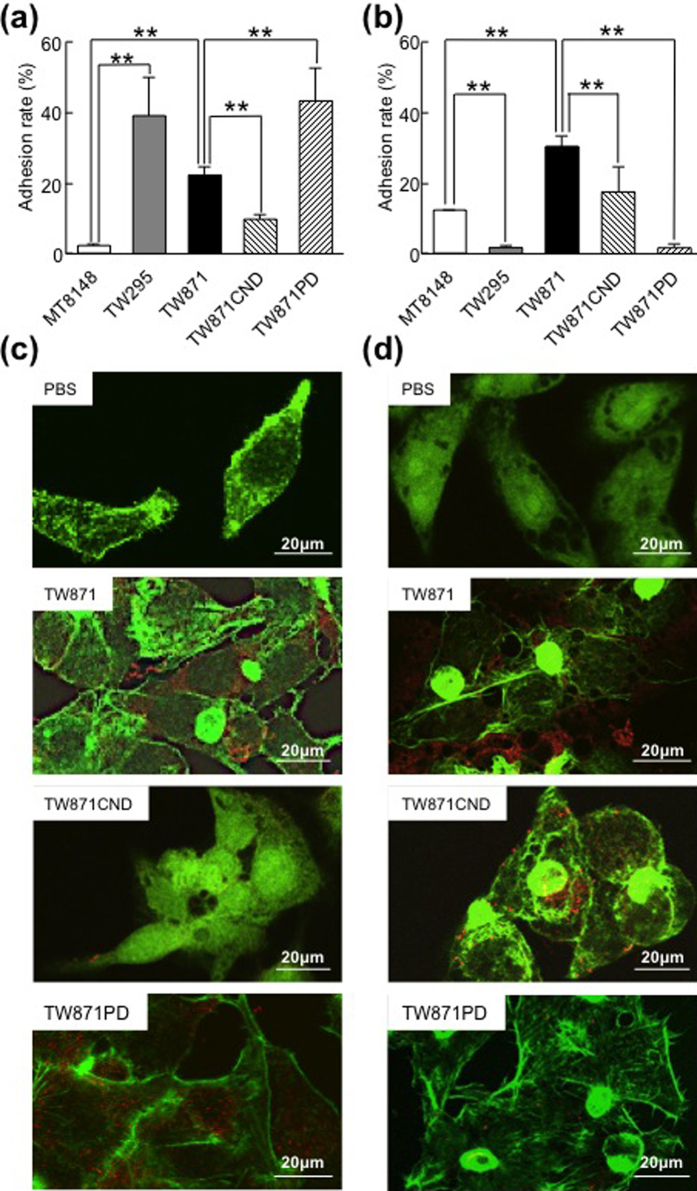
Bacterial adhesion to cultured hepatic cells with or without oleic acid. (**a**,**b**) Adhesion rates of *S. mutans* strains to HepG2 cells without (**a**) and with (**b**) oleic acid were measured. The adhesion rate was calculated as follows.: [(Number of the detected bacterial cells/Number of bacterial cells initially added to the HepG2 cells) × 100 (%)]. The results are shown as the mean ± SD from three independent experiments. Statistical significance was determined using Bonferroni’s method after ANOVA. **p < 0.01. (**c,d**) Representative confocal laser microscopy images of *S. mutans* strains attached to HepG2 cells without (**c**) and with (**d**) oleic acid. The red dots indicate *S. mutans* colonies.

The results of our adhesion assays using HepG2 and bacterial cells in the absence of oleic acid demonstrate that the Cnm^+^ strains TW295 and TW871 had significantly higher adhesion rates to HepG2 cells under these conditions than did the Cnm^−^ strain MT8148 (*p* < 0.05), while the adhesion rate of another Cnm^−^ strain, TW871CND, was significantly lower than those of the Cnm^+^ strains TW871 and TW871PD (*p* < 0.01) (Fig. 6a). However, the adhesion rate of TW871PD was significantly higher than that of TW871 (*p* < 0.01). In contrast, when oleic acid was added, the PA^+^ strains MT8148 and TW871 showed higher rates of adhesion to HepG2 cells than did the PA^−^ strain TW295 (*p* < 0.01), and the adhesion rate of another PA^−^ strain, TW871PD, was significantly lower than those of the PA^+^ strains TW871 and TW871CND (*p* < 0.01) (Fig. 6b). Confocal laser scanning microscopy observations demonstrated that there was a higher level of bacterial adhesion to HepG2 cells when they were incubated with TW871 group than when they were incubated with TW871CND (Fig. 6c). Furthermore, with the addition of oleic acid, the level of bacterial adhesion was much higher in HepG2 cells incubated with TW871 than that in the cells incubated with TW871PD (Fig. 6d). When analysing the bacterial adhesion rates in the presence of linoleic acid, the results were shown to be different than those in the presence of oleic acid (Fig. S2).

## Discussion

We previously reported that intravenous administration of *S. mutans* TW871 aggravates NASH conditions^7^, though the relevant mechanism remained to be elucidated. In the present study, we analysed the virulence of formalin-treated TW871 in a NASH mouse model. The TW871 that had been treated with formalin did not induce NASH aggravation (Fig. S3), suggesting that viable cells may be crucial for the NASH aggravation caused by *S. mutans* TW871. Next, we attempted to describe the molecular basis for that aggravation by focusing on two major cell surface protein antigens, Cnm and PA, both of which are expressed by TW871. The results from our animal experiments show that isogenic mutant strains of TW871 defective in Cnm (TW871CND) or PA (TW871PD) did not aggravate NASH conditions, whereas infection with either of the complemented strains (TW871CNDcomp and TW871PDcomp) reproduced the aggravated NASH conditions, suggesting these cell surface protein antigens are important for *S. mutans*-related aggravation of NASH.

The bacterial clearance assay in our NASH mouse model revealed that TW871 was cleared within 6 h; therefore, this defines the period in which the bacteria may influence the pathogenesis of NASH aggravation. Additionally, the defects of Cnm or PA in TW871 resulted in an enhanced bacterial clearance of the liver compared with that of TW871, which may contribute to the impaired NASH aggravation seen in mice infected with TW871CND or TW871PD. Furthermore, our systemic dissemination assessment of TW871 revealed a liver tropism, although the bacteria were also frequently isolated from spleen, indicating that the liver is one of the major organs targeted by *S. mutans* after it has invaded the bloodstream (Table S1). Thus, we decided to conduct *in vitro* studies to investigate the interaction of *S. mutans* strains with liver tissue.

Cnm is associated with the virulence of infective endocarditis caused by adhesion and invasion of endothelial cells by bacteria^3^. Here, the Cnm^+^ strains TW295 and TW871 showed significantly higher rates of adhesion to cultured hepatic cells than the Cnm^−^ strains TW871CND and MT8148. Thus, we suggest that Cnm is an important protein antigen related to *S. mutans*-associated NASH aggravation because of its contribution to adherence to hepatic cells without fatty acid accumulation. Notably, a comparable level of binding activity in cultured hepatic cells to those of TW871CND and MT8148 was observed in the Cnm^−^/PA^−^ double knockout TW871CNPD (Fig. S4), the presence of a similar amount of residual binding in the double knockout as in the strains that lack only Cnm suggests that an additional adhesin(s) may exist. This possibility should be investigated in future studies.

The cell surface protein antigen PA is known to be associated with cellular hydrophobicity^6^, indicating the possibility of it also having a relationship with the cellular incorporation of fatty acids. The present analysis of bacterial cell incorporation of unsaturated fatty acids demonstrates that PA^+^ strains, such as MT8148 and TW871, have significantly higher levels of fatty acid incorporation than the PA^−^ strain TW295. Additionally, our observations regarding the PA-defective isogenic mutant strain TW871PD demonstrate that it has a significantly lower fatty acid incorporation rate than that of the parent strain TW871. It is of interest that this was seen only in the presence of unsaturated, but not saturated, fatty acids. Thus, this suggests that excess unsaturated fatty acid intake can accelerate fat accumulation in *S. mutans*-related NASH aggravation. Additionally, TW871PD bound to hepatocytes at a significantly higher level than the parent strain, suggesting that PA may partially mask Cnm on the surface or that inactivation of PA may induce an upregulation in the expression of Cnm or an alternative adhesin(s).

Notably, oleic acid significantly impairs Cnm-mediated adhesion to hepatocytes, whereas adhesion mediated by PA or an alternative adhesin(s) is seemingly promoted by oleic acid. As the mice in our experiments were fed a diet in which the fat content is 76.9% unsaturated fats, it is likely that their livers contained some amount of unsaturated fat. The analyses of Nile blue staining of the livers from the mice after 4 weeks of consuming a high-fat diet demonstrate that fatty acid accumulation occurred in some areas (Fig. S5). We speculate that Cnm and PA are advantageous for the adhesion of *S. mutans* cells to hepatic cells without and with fatty acids, respectively. Therefore, strains that contain both antigens are advantageous for the aggravation of NASH as a second hit in the so-called “two-hit theory”. Further investigations should be performed to clarify the details of the mechanisms responsible for these effects.

PA is known to be an important factor in sucrose-independent initial adhesion to tooth surfaces^6^. Thus, *S. mutans* strains exhibiting PA expression are commonly detected in samples obtained from the oral cavity, with a prevalence of greater than 90%^18^. In contrast, it was reported that the distribution rate of Cnm^+^ *S. mutans* strains in the oral cavity is only 10%, among which PA^+^ strains comprise 75%^3^. Therefore, the distribution of Cnm^+^/PA^+^ *S. mutans* strains in the general population is estimated to be approximately 7.5%, and those individuals harbouring these strains may be at greater risk for *S. mutans*-associated aggravation of NASH. A large-scale survey regarding the distribution of Cnm^+^/PA^+^ *S. mutans* strains in the general population as well as in the subset of NASH patients should be performed.

The present study demonstrated that Cnm^+^/PA^+^ *S. mutans* strains are highly virulent for the aggravation of NASH. In contrast, Cnm^+^/PA^−^ *S. mutans* strains have been shown to be highly virulent for the aggravation of infective endocarditis and cerebral haemorrhage^4^,^19^. PA is known to be associated with cellular hydrophobicity^6^, indicating that its presence is advantageous for interaction with fatty acids. In fact, the present study found that PA interacted with the unsaturated fatty acids oleic acid and linoleic acids, but did not interact with the saturated fatty acids palmitic acid and tripalmitic acid. This suggests that PA^+^ strains are particularly good at incorporating oleic and linoleic acids. Thus, we focused on the incorporation of oleic acid by PA^+^ strains in the present study. In contrast, a defect in PA was reported to lower the bacterial susceptibility to phagocytosis by polymorphonuclear leukocytes, which results in a longer duration of bacteremia^18^ and may be advantageous for the development of cardiovascular diseases. Therefore, when identifying Cnm^+^ strains, it might also be important to confirm the expression of PA.

Based on our previous microarray analysis results, the expression levels of genes encoding cytokines, such as metallothionein and IFNγ, are elevated in *S. mutans*-related NASH aggravation in a mouse model^7^. Metallothionein is generally expressed in liver and kidney tissues, and its overexpression causes oxidative stress and generates reactive oxygen^20^, whereas overexpression of IFNγ could be associated with mechanisms of NASH aggravation^21^. Here, our immunochemical evaluation detected the prominent expression of these two molecules in liver tissues from the TW871-infected group, in contrast with their expression in the groups infected with other *S. mutans* strains. Therefore, Cnm^+^/PA^+^*S. mutans* strains may cause aggravation of NASH by elevating the expression of metallothionein and IFNγ in liver tissues.

It is important to consider whether or not the 1 × 10^7^ CFU administered to the mice in the present NASH mouse model accurately represent the *S. mutans* bolus that enters the bloodstream after a dental procedure. Unfortunately, there are currently no available data concerning the average amount of bacteria involved in this scenario. Our preliminary experiment demonstrated that the administration of 1 × 10^8^ CFU of *S. mutans* to the mice in this NASH model resulted in death, whereas that of 1 × 10^6^ CFU or less did not produce clear findings related to NASH aggravation; this led us to set the bacterial amount administered to this mouse model as 1 × 10^7^ CFU. However, it is estimated that even 1 mg of dental plaque contains more than 1 × 10^7^ bacteria^22^, suggesting that it is possible for such an amount of bacteria to enter the bloodstream when an invasive dental treatment is performed. Further studies should be conducted on how many bacteria can enter the bloodstream during each dental procedure.

**Figure 7:**
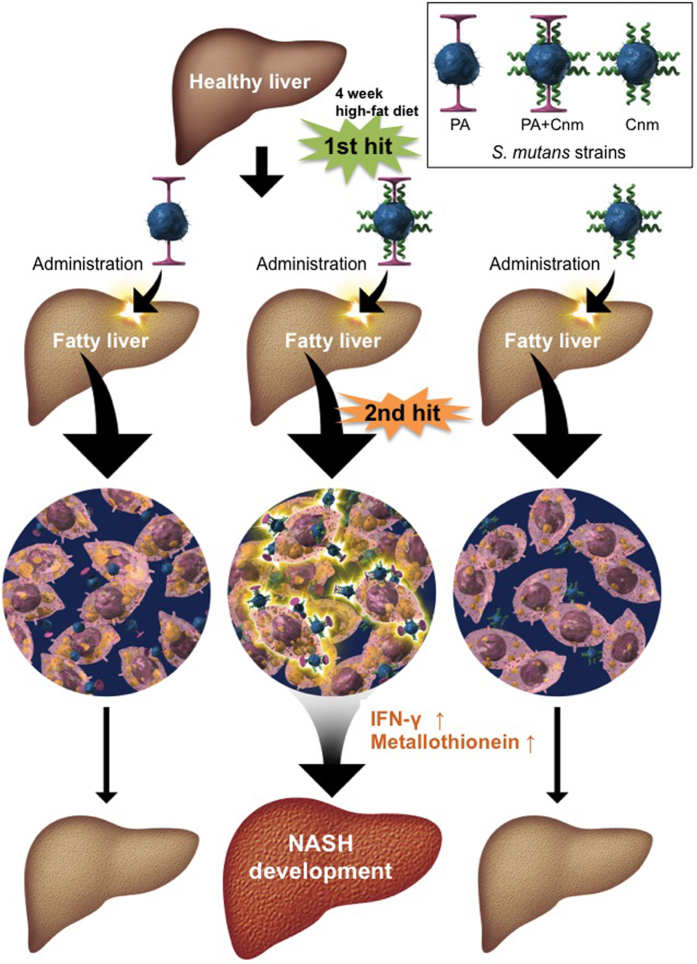
Model of our suggested mechanism for *S. mutans*–associated aggravation of NASH in mice. *S. mutans* organisms expressing both PA and Cnm on the surface of their bacterial cells are localised in the liver and attach to hepatocytes containing fatty acids. This induces elevated levels of inflammatory cytokines associated with oxidative stress, such as IFNγ and metallothionein, and may be regarded as the second hit of the “two-hit theory” in the aggravation of NASH. This phenomenon seems to only be induced by strains with both Cnm and PA expression on the bacterial cell surface; Cnm-defective and/or PA-defective strains do not appear to aggravate NASH in mice.

In summary, we identified *S. mutans* organisms expressing both Cnm and PA on the surface of their bacterial cells localised in the liver attached to hepatocytes in the presence of fatty acids. This localisation and attachment of the bacterial cells in the liver tissue may be one of the direct or indirect factors that induce the elevated levels of inflammatory cytokines associated with oxidative stress, such as IFNγ and metallothionein, and may be regarded as the second hit of the two-hit theory in the aggravation of NASH (Fig. 7). Based on our present results, it is possible that this phenomenon is only induced by strains wit both Cnm and PA expression on the bacterial cell surface and cannot be induced by Cnm-defective or PA-defective strains. Further large-scale studies with human subjects are required to validate our findings.

## Methods

### *S. mutans* strains

Table 1 lists the *S. mutans* strains used in the present study. *S. mutans* MT8148 (serotype *c*) is routinely used as a type strain^23^ and was isolated from the oral cavity of a Japanese child. TW295 (serotype *k*), isolated from the blood of a patient with bacteraemia after tooth extraction, and TW871 (serotype *k*), isolated from the blood of a patient with infective endocarditis complicated with subarachnoid haemorrhage, were also used^24^, along with the Cnm-defective isogenic mutant strain TW871CND^25^ and PA-defective isogenic mutant strain TW871PD^3^. Additionally, complemented mutant strains of TW871CND (TW871CNDcomp) and TW871PD (TW871PDcomp) were generated by a previously described method (Figs S6 and S7). Furthermore, a strain lacking both Cnm and PA was also generated by a previously described method (Fig. S8). All strains were cultured on Mitis-Salivarius (MS) agar (Difco Laboratories, Detroit, MI, USA) plates containing bacitracin (0.2 U/ml; Sigma Chemical Co., St. Louis, MO, USA) and 15% (wt/vol) sucrose (MSB agar) or brain heart infusion (BHI; Difco) broth. When culturing strains TW871CND, TW871PD, TW871PDcomp, TW871CNDcomp, and TW871CNPD, either erythromycin only, or erythromycin along with spectinomycin or kanamycin (Table 1) was added to the MSB agar (10 μg/ml) or BHI broth (10 μg/ml).

### Animal experiments

All mice were treated humanely in accordance with National Institute of Health and AERI-BBRI Animal Care and Use Committee guidelines. All procedures used in the present study were approved by the Animal Care and Use Committee of the Osaka University Graduate School of Dentistry. The effects of intravenous administrations of *S. mutans* strains on the development of NASH were analysed using a NASH mouse model as previously described^26^, with some modifications^7^. Briefly, 60 C57BL/6 J male mice (6 weeks old, Charles River Japan, Tokyo, Japan) were randomly divided into the high-fat diet (HFD) control, HFD plus TW871 administration, and HFD plus TW871PD or TW871CND administration groups (Experiment 1). Mice were allowed free access to water and food throughout the experimental period. High-fat diet 32 (Japan CLEA, Tokyo, Japan), which contains 506.8 kcal/100 g (57.5% from fat, 19.7% from protein, and 22.8% from carbohydrate), was used to feed the mice in the HFD groups. The levels of the saturated and unsaturated fatty acids in the HFD were 22.3% and 76.9%, respectively. Four weeks after beginning HFD ingestion, 1 × 10^7^ CFU of the *S. mutans* strains suspended in 100 μl of PBS or PBS without the added bacteria was intravenously injected via the jugular vein. Twelve weeks after infection (16 weeks after beginning HFD ingestion), the mice were euthanised, and whole body, extirpated liver tissue, and visceral fat weights were measured. Serum levels of AST and ALT were also measured by the laboratory of FALCO Biosystems Ltd. (Kyoto, Japan). The results are expressed as the mean ± standard error of the mean (SEM) from 11–17 different animals. Statistical significance was determined using Bonferroni’s method after analysis of variance (ANOVA). Tissue samples were fixed in 3.7% formaldehyde in PBS, embedded in paraffin, and cut into 3-μm sections for histopathological analysis. Haematoxylin-eosin staining was performed to evaluate the infiltration of inflammatory cells and adipocellular deposition, while Masson’s trichrome staining was used to evaluate fibrotic conditions. Additionally, alterations in IFNγ and metallothionein expressions in the tissues were detected using standard immunohistochemical techniques with IFNγ- and metallothionein-specific antibodies, respectively^5^. A Vectastain ABC kit and a 3, 3′-diaminobenzidine Substrate kit (Vector Laboratories, Burlingame, CA, USA) were used for immunochemical analyses according to the manufacturer’s instructions. The same experiment was then performed using groups of mice infected with the strains TW871, TW871CND, TW871CNDcomp, TW871PD, TW871PDcomp, and TW871CNPD (Experiment 2).

### Detection of bacteria in tissue samples

Bacterial detection was performed using a previously described method^5^ with some modifications. Briefly, liver and blood specimens were obtained from mice just before and 1, 3, or 6 h after injection of 1 × 10^7^ CFU of *S. mutans* TW871 into the jugular vein. Additionally, liver and blood specimens were obtained just before and after 1, 3, and 6 h. Additionally, liver, blood, heart, lung, spleen, pancreas, kidney, and colon specimens were obtained after 1 h. All of these tissue samples were cut into small pieces and homogenised, and the resultant liquid was serially diluted with sterile PBS, placed onto MSB agar plates, and anaerobically incubated at 37 °C for 48 h. The blood specimens were obtained using heparinised plastic tubes, serially diluted with sterile PBS, and placed onto MSB agar plates and anaerobically incubated at 37 °C for 48 h. The number of bacterial colonies was then counted by visual inspection of the plates, which was standardised by the liver weight (g) or blood amount (ml).

To confirm that the recovered bacteria in the liver and blood were *S. mutans*, the following PCR technique was performed. Bacterial DNA was extracted from the extirpated tissues and then subjected to PCR using an *S. mutans*-specific sets of primers (5′-GGC ACC ACA TTG GGA AGC TCA GTT-3′ and 5′-GGA ATG GCC GCT AAG TCA ACA GGA T-3′)^27^. PCR amplification was performed in a total volume of 20 μl with 1 μl of template solution and *Takara Ex Taq*^®^ (Takara Bio Inc., Otsu, Japan), according to the supplier’s instructions. The PCR amplification reaction was performed in a thermal cycler (iCycler; Bio-Rad, Hercules, CA, USA) with the following cycling parameters: 30 cycles of a denaturing step at 98 °C for 10 s, and a primer-annealing and extension step at 70 °C for 1 min. The PCR products were subjected to electrophoresis in a 1.5% agarose gel with Tris-acetate-EDTA buffer, and the gel was stained with ethidium bromide (0.5 μg/ml) and photographed under UV illumination. A 100-bp DNA ladder (New England BioLabs, MA, USA) was used as the molecular size standard.

### Affinity of cultured hepatic cells for saturated and unsaturated fatty acids

The affinity of the cultured hepatic cells for saturated and unsaturated fatty acids was evaluated by the methods previously described by Gómez-Lechón *et al*.^28^ and Hozumi *et al*.^29^ with some modifications. First, 1 mM of each fatty acid was incubated for 24 h with the cultured hepatic cells (HepG2 cells derived from human hepatic cancer cells; DS Pharma Biomedical Co., Ltd., Osaka, Japan) in Dulbecco’s modified Eagle’s Medium (DMEM) (Sigma-Aldrich, Tokyo, Japan) containing foetal bovine serum (FBS) (Nichirei Biosciences Inc., Tokyo, Japan), which is lipid- and fatty acid-free, for 24 h. The mixtures were then washed twice with PBS, fixed with 10% formalin for 10 min at the room temperature, and then washed twice more. Next, 1 ml of 60% isopropanol was added and incubated on the samples for 1 min before being discarded, after which 1 ml of Oil Red O staining solution was added and incubated with the samples for 20 min at room temperature. Final washes were conducted, using 60% isopropanol once, followed by PBS twice. Finally, the culture dishes were examined by light microscopy (Inverted Microscope Olympus CKX41, Olympus, Osaka, Japan).

### Bacterial incorporation of fatty acids

Because fatty acid incorporation by bacteria may be involved in the bacterial attachment to fatty liver tissue, the bacterial incorporation of saturated and unsaturated fatty acids was evaluated using the method for the cellular hydrophobicity assay constructed by Rosenberg *et al*.^15^ with some modifications. Briefly, each test strain was cultured in 40 ml of BHI broth and collected by centrifugation. The cells were washed three times and suspended in saline to an OD_550_ value of 0.6. Then, 0.2 ml of n-hexadecane (Hexadecane; Wako, Osaka, Japan), the unsaturated fatty acids oleic acid (Wako) and linoleic acid (Wako), or the saturated fatty acids palmitic acid (Wako) and tripalmitin (Wako) were added to the bacterial cells (3 ml), and they were uniformly agitated with a vortex mixer for 1 min. The mixture was left to stand for 10 min at room temperature. After separating the aqueous phase from the phase containing n-hexadecane or a fatty acid, the optical density of the aqueous phase was determined at OD_550_. The incorporation rate was calculated as follows: [1 − OD_550_ (aqueous phase of the tube containing cell suspensions with added n-hexadecane or one of the tested fatty acids)/OD_550_ (aqueous phase of the tube containing only cell suspensions)] × 100 (%)]. The results are shown as the mean ± SD from five independent experiments. The statistical significance of differences between groups was determined using Bonferroni’s method after ANOVA.

### Bacterial adhesion to cultured hepatic cells with or without unsaturated fatty acids

The bacterial incorporation into fatty liver tissue, which may contain high levels of fatty acids, was estimated using cultured hepatic cells in a previously described method^5^ with some modifications. Briefly, approximately 1 × 10^5^ HepG2 cells were seeded in parallel wells of 24-well tissue-culture plates. Prior to infection, the wells were washed three times with PBS, and antibiotic-free medium was added. The HepG2 cells were infected by the addition of 1 × 10^7^ CFU of each bacterial strain with or without the interaction inclusion of oleic acid. After 10 h of aerobic incubation, the medium was removed, and the infected cells were washed three times with PBS. To test for adherence, 1.0 ml of sterile distilled water was added, and the cultured cells were allowed to burst. Various dilutions of the lysates of *S. mutans*-infected cells were plated on MSB agar and cultured at 37 °C for 48 h under anaerobic conditions. The adhesion rate was calculated as follows: [(Number of the detected bacterial cells/Number of bacterial cells initially added to the HepG2 cells) × 100 (%)]. The number of adherent bacteria was determined and expressed as the mean ± SD of triplicate experiments. Statistical significance was determined using Bonferroni’s method after ANOVA.

To visually confirm bacterial adhesion, a previously described double-fluorescence technique^3^ for observation with confocal scanning laser microscopy was utilized with some modifications. Briefly, the cells were then fixed with 4% paraformaldehyde (Wako Chemical Industries) for 10 min, washed with PBS, and incubated for 1 h at room temperature with rabbit anti-*S. mutans* Cnm serum^30^, or with rabbit anti-*S. mutans* glucosyltransferase D serum^31^ for the knockout strains, diluted 1:500 with PBS containing 0.5% bovine serum albumin. Following incubation, the dishes were washed three times with PBS and incubated for 30 min at room temperature with Alexa Fluor 633-conjugated goat anti-rabbit immunoglobulin G (Molecular Probes^®^; Life Technologies Co., Eugene, OR, USA). Actin filaments were stained with Alexa Fluor 488-conjugated to phalloidin (Molecular Probes^®^; Life Technologies Co.) for 30 min at room temperature to visualise the cellular skeleton. Culture dishes were examined by confocal scanning laser microscopy using a TCS-SP5 microscope (Leica Microsystems GmbH, Wetzlar, Germany) and a DMI1600 B fluorescence microscope (Leica) equipped with a 63× oil immersion objective.

### Statistical analyses

Statistical analyses were performed using WinSTAT Statistics Software, and *p*-values of less than 0.05 were considered to be significant.

## Additional Information

**How to cite this article**: Naka, S. *et al*. Contributions of *Streptococcus mutans* Cnm and PA antigens to aggravation of non-alcoholic steatohepatitis in mice. *Sci. Rep.* **6**, 36886; doi: 10.1038/srep36886 (2016).

**Publisher’s note:** Springer Nature remains neutral with regard to jurisdictional claims in published maps and institutional affiliations.

## Supplementary Material

Supplementary Information

## Figures and Tables

**Table 1:** *S. mutans* strains used in the present study.

Strains^a^	Cell surface protein expression	Features^b^	References
MT8148 (*c*)	Cnm (−) PA (+)	Oral isolate from a Japanese child; Standard strain	Ooshima *et al*.^23^
TW295 (*k*)	Cnm (+) PA (−)	Blood isolate from a Japanese patient with bacteremia after tooth extraction	Nakano *et al*.^24^
TW871 (*k*)	Cnm (+) PA (+)	Blood isolate from a Japanese patient with infective endocarditis complicated with subarachnoid haemorrhage	Nakano *et al*.^24^
TW871CND (*k*)	Cnm (−) PA (+)	Em^r^; Cnm-defective isogenic mutant of TW871	Nakano *et al*.^25^
TW871CNDcomp (*k*)	Cnm (+) PA (+)	Em^r^ Spe^r^; Complemented mutant of TW871CND	This study
TW871PD (*k*)	Cnm (+) PA (−)	Em^r^; PA-defective isogenic mutant of TW871	Nomura *et al*.^3^
TW871PDcomp (*k*)	Cnm (+) PA (+)	Em^r^ Spe^r^; Complemented mutant of TW871PD	This study
TW871CNPD (*k*)	Cnm (−) PA (−)	Em^r^ Km^r^; Cnm- and PA-defective isogenic mutant of TW871	This study

^a^The serotype of each strain is indicated in parentheses.

^b^Em^r^: erythromycin-resistant, Spe^r^: spectinomycin-resistant, Km^r^: kanamycin-resistant.
