# Supplementary material for: Contributions of Streptococcus mutans Cnm and PA antigens to aggravation of non-alcoholic steatohepatitis in mice
Source: Sci Rep. 2016 Nov 11;6:36886. doi: 10.1038/srep36886 (PMC5105074; doi:10.1038/srep36886)
Supplement: Supplementary Information [file srep36886-s1.docx]

**Supplementary Information**

**Contributions of *Streptococcus mutans* Cnm and PA antigens to aggravation of non-alcoholic steatohepatitis in mice**

Shuhei Naka^1^, Rina Hatakeyama^1^, Yukiko Takashima^2^, Michiyo Matsumoto-Nakano^2^, Ryota Nomura^1^, and Kazuhiko Nakano^1^

^1^Department of Pediatric Dentistry, Division of Oral Infection and Disease Control, Osaka University Graduate School of Dentistry, Suita, Osaka, Japan

^2^ Department of Pediatric Dentistry, Okayama University Graduate School of Medicine, Dentistry and Pharmaceutical Sciences, Okayama, Japan

**Correspondence should be addressed to:**

Kazuhiko Nakano, DDS, PhD., Department of Pediatric Dentistry, Osaka University Graduate School of Dentistry, 1-8 Yamada-oka, Suita, Osaka 565-0871, Japan

E-mail: [nakano@dent.osaka-u.ac.jp](mailto:nakano@dent.osaka-u.ac.jp); Fax: +81-6-6879-2965

**Supplementary information includes:**

Supplementary Table S1, Supplementary Figures S1 to S8.

**Supplementary Tables**

**Supplementary Table S1.** Bacterial recovery from extirpated organs of mice 1 h after infection with *S. mutans* TW871.

|  |  |
| --- | --- |
| Extirpated tissues | Number of mice with bacterial recovery |
|  |  |
|  |  |
| Liver | 5/5 |
|  |  |
| Blood | 5/5 |
|  |  |
| Spleen | 5/5 |
|  |  |
| Heart | 2/5 |
|  |  |
| Kidney | 2/5 |
|  |  |
| Lung | 0/5 |
|  |  |
| Pancreas | 0/5 |
|  |  |
| Colon | 0/5 |
|  |  |

**Supplementary Figures**


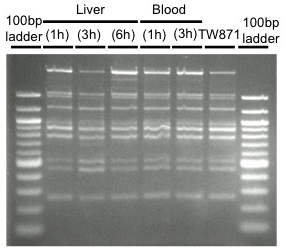


**Supplementary Fig. S1.** Comparison of DNA fingerprinting patterns by the AP-PCR analyses of the administered and recovered bacteria.

The fingerprinting patterns of the recovered strains were compared using an arbitrarily primed (AP)-PCR method with the OPA-13 primer (5ʹ-TGC CGA GCT G-3ʹ), as described by Li & Caufield (1998). The PCR assay included 45 cycles of denaturing at 94 °C for 30 s, annealing at 36 °C for 30 s, and extension at 72 °C for 1 min. Amplicons were separated by electrophoresis on 1.5% agarose gels.

**Reference**

Li, Y. & Caufield, P.W. Arbitrarily primed polymerase chain reaction fingerprinting for the genotypic identification of mutans streptococci from humans. *Oral Microbiol. Immunol.* **12,** 17–22 (1998).


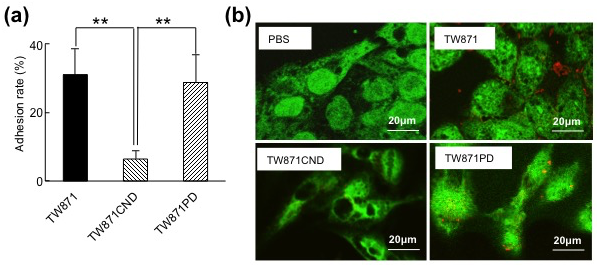


**Supplementary Fig. S2.** Bacterial adhesion to cultured hepatic cells in the presence of linoleic acid.

(a) Adhesion rates of *S. mutans* strains to HepG2 cells in the presence of linoleic acid were measured as described in the Methods section. The results are shown as the mean ± SD from three independent experiments. Statistical significance was determined using Bonferroni’s method after ANOVA. ***p* < 0.01. (b) Representative confocal laser microscopy images of *S. mutans* strains attached to HepG2 cells in the presence of linoleic acid. The red staining indicates *S. mutans* colonies.


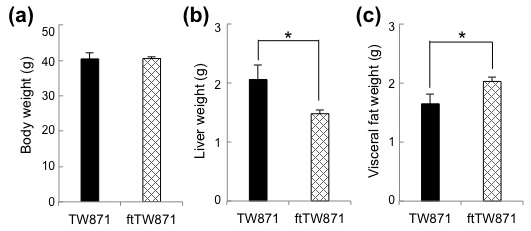


**Supplementary Fig. S3.** Evaluation of formalin-treated TW871 in regard to aggravation of NASH in a mouse model.

After feeding 6-week-old, male, C57BL/6J mice with a high-fat diet for 4 weeks, the mice were infected with 1×10^7^ CFU of *S. mutans* TW871 or formalin-treated TW871 (ftTW871), which was generated by washing the TW871 with PBS and then incubating them in formalin (Wako, Osaka, Japan) for 16 h at 4 °C. Twelve weeks after infection, the mice were euthanised (16 weeks after initiating the HFD), and the NASH characteristics were evaluated. Body (a), liver (b), and visceral fat (c) weights are shown for each group. Each column represents the mean ± SEM from seven or eight different animals. Statistical significance was determined using Student’s *t*-test. **p* < 0.05.

**
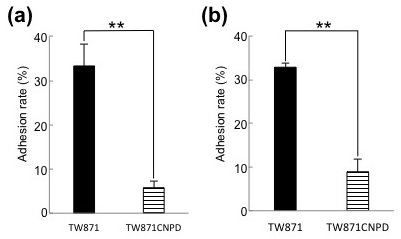
**

**Supplementary Fig. S4.** Bacterial adhesion of a Cnm- and PA-defective TW871 mutant to cultured hepatic cells.

The adhesion of a Cnm- and PA-defective double knockout mutant of TW871 (TW871CNPD) to cultured hepatic cells in the presence or absence of oleic acid was assessed as described in the Methods section. Adhesion rates of *S. mutans* strains to HepG2 cells in the absence (a) or presence (b) of oleic acid were measured. The results are shown as the mean ± SD from three independent experiments. Statistical significance was determined using Student’s *t*-test. ***p* < 0.01.

**
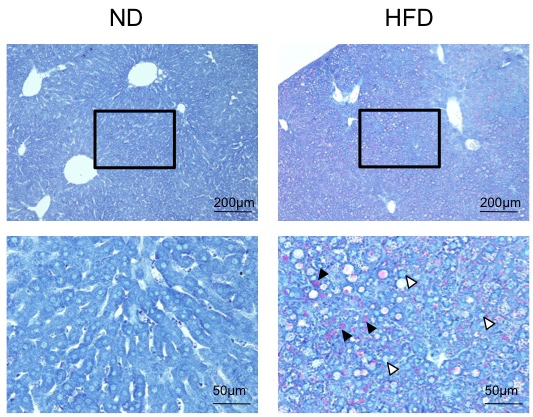
**

**Supplementary Fig. S5.** Histopathological appearance of liver tissue extirpated just before administration of *S. mutans* strains.

C57BL/6J male mice were fed with a normal diet (CE-2; Japan CLEA, Tokyo, Japan) or a high-fat diet (HFD) for 4 weeks (n = 5). The mice then were euthanised, followed by extirpation of the liver and generation of histopathological sections, which were stained with Nile blue. Representative images from livers of mice fed a normal dies (ND; left) and of those fed the HFD (right) are shown. The lower images are a magnification of rectangular space in the upper images. The red (marked by black arrowheads) and blue areas (marked by white arrowheads) indicate the presence of neutral fat and fatty acids, respectively (Cañete *et al*., 1983).

**References**

Cañete, M., Hazen, M. J. & Stockhert, J. C. Nile blue sulfate staining for demonstration of lipids in fluolescence microscopy. *Acta. Histochem. Cytochem.* **16,** 286-288 (1983).

**
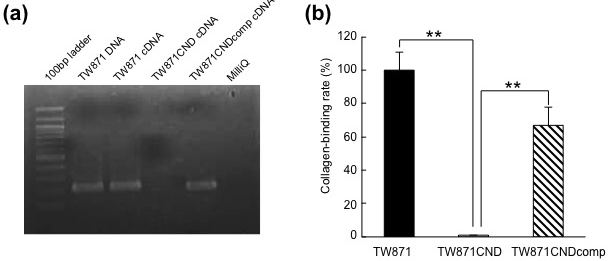
**

**Supplementary Fig. S6.** Construction of complemented mutant strain of TW871CND (TW871CNDcomp). To generate TW871CNDcomp, TW871CND was transformed with plasmid pDL278 (Dunny *et al*., 1991) containing an intact copy of the corresponding deleted gene. Briefly, the entire *cnm* was amplified by PCR with TaKaRa ExTaq polymerase (Takara Bio, Inc., Otsu, Japan) using the primer set Forward 5ʹ-GAC AAA GAA ATG AAA GAT GT-3ʹ, and Reverse 5ʹ-GCA AAG ACT CTT GTC CCT GC -3ʹ, which incorporated an appropriate restriction site for subsequent cloning into pDL278. The amplified DNA fragment was purified with phenol chloroform and precipitated with ethanol. This fragment was then digested with *BamH* I and *Hind* III and cloned into pDL278. Transformation into TW871CND was performed according to the protocol of Lindler & Macrina (1986). Overnight cultures of TW871CND were inoculated into Todd–Hewitt medium supplemented with 10% heat-inactivated horse serum (Invitrogen, Carlsbad, CA, USA) and incubated for 2 h. Approximately 200 μg of the plasmid was added to growing liquid cultures, and the samples were incubated for 2 h at 37 °C. The cells were then collected by centrifugation, plated on MS agar containing spectinomycin (1 mg/ml), and incubated anaerobically at 37 °C for 48 h. One positive transformant, TW871CNDcomp, was selected, and the expression of its *cnm* gene was confirmed using reverse transcription PCR as described previously (Nomura *et al*., 2005) (a). First, total RNA was prepared from the tested strains using a FastPrep® Cell Disrupter (Model FP 100A, Q-Bio gene, Carlsbad, CA, USA) in combination with a Fast RNA® Pro Blue kit (Q-Bio gene), according to the manufacturer’s instructions. SuperScript™ III Reverse Transcriptase (Invitrogen) was used to amplify the cDNA synthesised from mRNA. The following primer set was designed based on the *cnm* sequence: Forward 5ʹ-CTA CCG GCG TTT TCT ACT ATA AGA CTG GGG-3ʹ, and Reverse 5ʹ-CCT TCT TGA CCG CGA TAA GAC TCA CTG CCA-3ʹ. The cDNA was transcribed from 100 ng of total RNA at 55 °C for 60 min and heated at 70 °C for 15 min. Successive PCR assays were performed under the following conditions: 30 cycles at 94 °C for 30 s, 54 °C for 30 s, and 72 °C for 30 s. Genomic DNA from TW871was used as a positive control. (b) A collagen-binding assay was then performed by the method described by Nomura *et al*. (2012)^30^. Briefly, type I collagen (2 mg/well of type I collagen (Sigma) in 0.25 M acetic acid) was coated onto 96-well tissue culture plates (Beckton Dickinson, Franklin Lakes, NJ, USA) and incubated overnight at 4 °C. The plates were then washed three times with PBS and blocked for 1.5 h with 5% bovine serum albumin in PBS at 37 °C. Next, the wells were washed again with PBS containing 0.01% Tween-20. Cells from overnight cultures of *S. mutans* grown in brain-heart infusion broth (Difco Laboratories, Detroit, MI, USA) were collected by centrifugation, and the bacterial cells were diluted with PBS and added to the wells (10^10^ colony-forming units per well). After incubation for 3 h at 37 °C, adherent cells were washed three times with PBS and fixed with 200μl of 25% formaldehyde at room temperature for 30 min. After another three washes with PBS, the adherent cells were stained with 200 μl of 0.05% crystal violet (Wako) in water for 1 min, and then washed three more times with PBS. The dye was subsequently dissolved by adding 200 μl of 7% acetic acid before determining the optical density at 595 nm. The results for each strain are expressed as a percentage relative to the binding ability of strain TW871, which was defined as 100%. Data are expressed as the mean ± standard deviation of triplicate experiments. Statistical significance was determined using Student’s *t*-test. ***p* < 0.001.

**References**

Dunny, G. M., Lee, L. N. & LeBlanc, D. J. Improved electroporation and cloning vector system for gram-positive bacteria. *Appl. Environ. Microbiol.* **57,** 1194-1201 (1991).

Lindler, L. E. & Macrina, F. L. Characterization of genetic transformation in *Streptococcus mutans* by using a novel high-efficiency plasmid marker rescue system. *J. Bacteriol.* **166,** 658-665, (1986).

Nomura, R., Nakano, K. & Ooshima, T. Molecular analysis of the genes involved in the biosynthesis of serotype specific polysaccharide in the novel serotype *k* strains of *Streptococcus mutans*. *Oral Microbiol. Immunol.* **20,** 303-309 (2005).

**
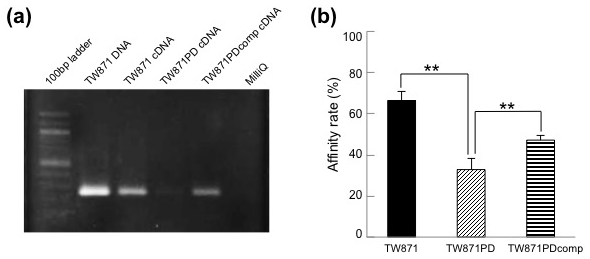
**

**Supplementary Fig. S7.** Construction of complemented mutant strain of TW871PD (TW871PDcomp). To generate TW871PDcomp, TW871PD was transformed with plasmid pDL278 (Dunny *et al*., 1991) containing an intact copy of the corresponding deleted gene. Briefly, the entire of *pac* was amplified by PCR with AmpliTaq (Life Technologies Co., Eugene, OR, USA) using the primer set Forward 5ʹ-GCG GCA TGC TTC AGA TTT GGA GGA TTT ATG-3ʹ and Reverse 5ʹ-AAT CCC GGG GTA ATA TCT ATG CTG TCA ATC-3ʹ, which incorporated an appropriate restriction site for subsequent cloning into pDL278. The amplified DNA fragment was purified with phenol chloroform and precipitated with ethanol. This fragment was digested with *Sph* I and *Xma* I, and subsequently cloned into pDL278. Transformation into TW871PD was carried out as described in Fig. S6. (a) One positive transformant, TW871PDcomp, was selected, and the expression of *pac* from TW871, TW871PD, and TW871comp was evaluated by reverse transcription PCR as described in Fig. S6, except that the following primer set designed based on the *pac* sequence was used instead: Forward 5ʹ-CCT AAA GGT GCT TTC CAA ATT TTC CGT GCC G-3ʹ and Reverse 5ʹ-CAG TCG GAT CAA GTG TTA AGG TCA CAT C-3ʹ. (b) The cellular hydrophobicity assay was performed as described in the Methods section. Statistical significance was determined using Student’s *t*-test. **p* < 0.05.

**References**

Dunny, G. M., Lee, L. N. & LeBlanc, D. J. Improved electroporation and cloning vector system for gram-positive bacteria. *Appl. Environ. Microbiol.* **57,** 1194-1201 (1991).

**
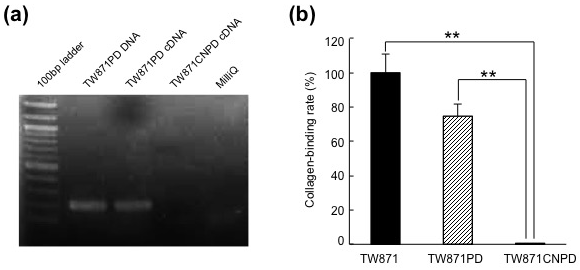
**

**Supplementary Fig. S8.** Construction of a double knockout mutant strain of TW871. The double knockout mutant strain of TW871 (TW871CNPD) was generated from TW871PD by insertional inactivation of *cnm*. Briefly, the *cnm* gene was amplified by PCR using Takara Ex Taq (Takara Bio Inc.) with primers constructed on the basis of the *cnm* sequence (Forward 5ʹ-GAC AAA GAA ATG AAA GAT GT-3ʹ and Reverse 5ʹ-GCA AAG ACT CTT GTC CCT GC-3ʹ). The amplified fragment was then cloned into a pGEM-T Easy vector (Promega Co., Madison, WI, USA) to generate pTN11 (Nakano *et al.*, 2010). The middle of *cnm* in pTN11 was cleaved by *Bsm*I, and then ligated with the kanamycin resistance gene (*aphA*) from transposon Tn1545 from the recombinant plasmid pKF5 (Matsumoto-Nakano *et al*., 2007). After linearisation by digestion at the unique restriction site of *Pst*I, the plasmid was introduced into *S. mutans* TW871PD using a homologous recombination method. The transformants were screened on MS agar plates containing kanamycin sulphate (500 µg/ml). The expression of *cnm* in each strain was confirmed by reverse transcription PCR as described in Fig. S6 (a). The collagen-binding assay was performed by the method described in Fig. S6 (b). Statistical significance was determined using Student’s *t*-test. ***p* < 0.001.

**References**

Matsumoto-Nakano, M., Fujita, K. & Ooshima, T. Comparison of glucan-binding proteins in cariogenicity of *Streptococcus mutans. Oral Microbiol. Immunol.* **22,** 30-35 (2007).

Nakano, K., *et al.* Molecular characterization of *Streptococcus mutans* strains containing the *cnm* gene encoding a collagen-binding adhesin. *Arch. Oral Biol.* **55,** 34-39 (2010).
